# Delay modulates the immune response to nerve repair

**DOI:** 10.1038/s41536-023-00285-4

**Published:** 2023-02-27

**Authors:** Masoud Golshadi, Elaine F. Claffey, Jennifer K. Grenier, Andrew Miller, Michael Willand, Michael G. Edwards, Tim P. Moore, Michael Sledziona, Tessa Gordon, Gregory H. Borschel, Jonathan Cheetham

**Affiliations:** 1 Cornell University College of Veterinary Medicine, 930 Campus Road, Ithaca, NY 14853 USA; 2 Epineuron Technologies Inc, 5100 Orbitor Dr., Mississauga, ON L4W 5R8 Canada; 3 Bioinfo Solutions LLC, Parker, CO USA; 4 Hospital for Sick Children, 555 University Ave, Toronto, ON M5G 1×8 Canada; 5 Indiana University, 107 S Indiana Ave, Bloomington, IN 47405 USA

**Keywords:** Somatic system, Immunology

## Abstract

Effective regeneration after peripheral nerve injury requires macrophage recruitment. We investigated the activation of remodeling pathways within the macrophage population when repair is delayed and identified alteration of key upstream regulators of the inflammatory response. We then targeted one of these regulators, using exogenous IL10 to manipulate the response to injury at the repair site. We demonstrate that this approach alters macrophage polarization, promotes macrophage recruitment, axon extension, neuromuscular junction formation, and increases the number of regenerating motor units reaching their target. We also demonstrate that this approach can rescue the effects of delayed nerve graft.

## Introduction

Peripheral nerve injury (PNI) following trauma leads to decreased function and long-term disability. Despite advances in microsurgical techniques, full recovery is seldom achieved. Repair may be delayed for several weeks or months to allow other soft tissue or orthopedic injuries to be addressed or, in some contexts, to allow the possibility of spontaneous recovery^1–5^. As the time between injury and repair increases, the proportion of patients experiencing a good recovery decreases dramatically^3,5,6^.

A prolonged delay before surgical reinnervation results in chronic denervation of the distal nerve stump and loss of the intimate association between Schwann Cells (SC) and their axons^2,6–8^. These effects contribute to poor regeneration when repair is delayed^2,7,9,10^. The most important contributing factor for poor recovery is the chronic denervation of axons distal to the injury site rather than the denervation of the target muscle^7,11^. As a consequence, delayed nerve repair in a clinical setting, results in progressive worsening of regeneration and recovery^1,3,5^.

Acute injury to the peripheral nervous system produces a well-orchestrated series of events that are required for successful regeneration and functional recovery^12–15^.

In the first three days after injury, resident macrophages and Schwann cells (SC) remove fragmented axonal segments (16–18) and the breakdown of the nerve-blood barrier allows macrophage migration and amplified phagocytosis to remove myelin-associated glycoprotein and other inhibitory proteins from the local environment^2,6,7,16^.

Macrophage migration into the nerve injury site, may be more important than the proliferation of resident macrophages in this process and occurs in response to immune mediators such as Interleukin-10 (IL10), IL6, and granulocyte macrophage colony-stimulating factor (GM-CSF), produced by resident SC^17,18^.

Macrophages promote the development of a polarized microvasculature, mediated by vascular endothelial growth factor (VEGF-A), which directs SC migration within the regenerative bridge which forms between the proximal and distal nerve stumps^19^. Regeneration does not occur when migrating macrophages are excluded from the site of injury^20,21^ and SC fail to proliferate and transdifferentiate. Conversely, increased macrophage recruitment can promote endothelial and SC migration and axon number^22^. In addition to VEGF, macrophages also secrete transforming growth factor-beta (TGFβ) and interleukin-1 (IL1), which promote the proliferation of SC and induce SC expression of nerve growth factor (NGF) which, in turn, promotes axonal growth^14,23–25^. In addition, macrophages can also affect regeneration by acting directly on axotomized cell bodies in the dorsal root ganglia^26^. Together, these data suggest that axonal regrowth, and so functional recovery, is very dependent upon the macrophage response to nerve injury.

Although macrophage recruitment to the injury site is essential for nerve repair, alterations in macrophage function after delayed repair have not been examined. In addition, the majority of attempts to improve outcomes after nerve graft have focused on changing events that occur late in the repair process. For example, the localized release of nerve-specific growth factors^27–32^ has been used to attempt to promote axon extension and conduits have been used to prevent infiltration of fibrous tissue and directly influence the repair process^33^.

Here, we build on our previous work examining temporal alterations in macrophage gene expression after nerve injury to examine this element of repair after a delay^34^. We first determined how delayed repair alters the immune response to PNI, and then examined the effect of delay on key macrophages functions including gene expression, phagocytosis, cytokine and protein production, and angiogenesis. We then identified a potential target for modulation of the macrophage response to delayed repair and demonstrate that this approach can alter downstream remodeling after nerve injury, altering macrophage gene expression, promoting axon extension, neuromuscular junction formation and the number of motor neurons reaching their targets. Overall, we show that targeted modulation of the macrophage response to delayed repair provides a strong opportunity for recovery after chronic denervation.

## Results

### Delayed repair after PNI results in a significant reduction of recovery

**Fig. 1:**
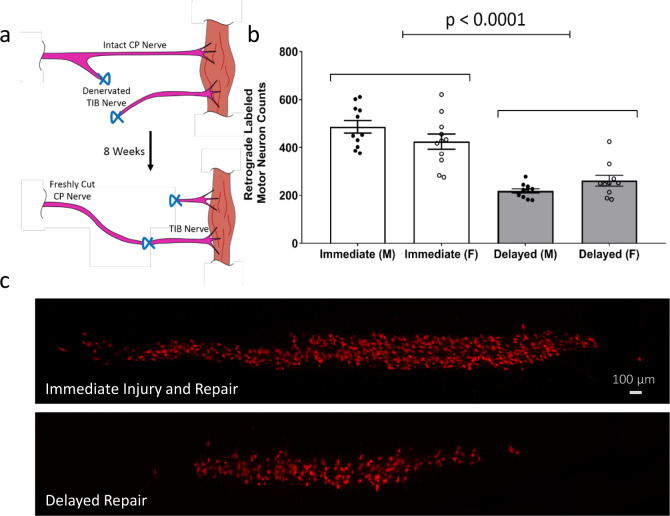
Delayed repair significantly reduces recovery after nerve graft. **a** Experimental paradigm. Freshly axotomized common peroneal (CP) nerve branch was grafted to the acutely or chronically denervated tibial (TIB). To mimic a delayed graft, the TIB nerve branch was transected 2–5 mm distal to the sciatic trifurcation and sutured to the quadriceps muscle. After 8 weeks of delay, the proximal (CP) and distal (TIB) stumps were aligned and cross sutured. Immediate repair was achieved with immediate CP-TIB cross-suture. **b** Retrograde tracer confirmed significantly lower number of regenerated motor neurons in the spinal cord after delayed repair compared to immediate injury and repair in both male and female animals (*P* < 0.0001, ANOVA, *n* = 11/group). No sex effect was observed. **c** Representative Z-stacks of mouse spinal cord (L4-S2) with retrograde-labeled motor neurons after delayed or immediate injury and repair.

We used a cross-suture repair experimental paradigm (Fig. 1a) in which a freshly transected common peroneal (CP) nerve branch is grafted to a distal tibial (TIB) stump either immediately or after a delay of eight weeks^11,16^. To allow appropriate comparison with the delayed repair group, the surgery site was incised (skin and muscle layers), the sciatic trunk exposed, and the incision then closed 8 weeks prior to nerve surgery in the immediate group. Repair was evaluated 8 weeks after CP-TIB cross-suture using retrograde labeling to determine the number of axons crossing the graft site and reaching their target^35^.

Sex has been shown to have an influence on recovery after PNI with an increase in the rate of axonal extension in female rats following crush injury and an increased regenerative response in males to treadmill exercise^36,37^. However, we identified no effect of sex on the reduction in reinnervation observed after delayed repair and confirmed a significant reduction in the number of regenerated motor neurons following delayed repair (Fig. 1b, c).

### Delayed repair after PNI downregulates and delays the immune response

We then determined transcription changes in macrophages and SC following chronic denervation by isolating macrophages and SCs from the site of immediate or delayed nerve graft by using S100β-eGFP mice expressing enhanced GFP (eGFP) in their SCs (hereafter S100) combined with a validated FACS approach which obviates the need for post isolation culture, avoiding alterations in macrophage or SC gene expression in vitro^38^. Cells were isolated at 5 and 14 days after repair, from silicone conduits inserted at the site of cross-suture. B cells, T cells, granulocytes, eosinophils, fibroblasts, endothelial cells, and red blood cells were excluded from the viable single-cell population (dump). Macrophages (CD16/32hi, CD11b hi, F4/80 hi, and CD14 hi) and Schwann cells (GFP hi and negative for all other markers) were isolated. The TIB stump was also removed (day 5 only) and RNA isolated from all three cell/tissue types. We choose these time points specifically to represent the early peak of macrophage infiltration^19,39,40^ and SC migration and dedifferentiation/activation^20,41,42^ and the later macrophage and Schwann cell responses.

Using this approach, we characterized the in vivo macrophage and SC transcriptomes by high throughput mRNA sequencing. Libraries were prepared from 10 biological replicates per group (immediate and delayed) at both 5 and 14 days after repair (total of 40 animals), generating an average of around 1 million reads per sample for the quantification of 23,361 genes. After confirming the quality and reproducibility of the RNA-Seq data, we examined differences in differentially expressed genes between cell/tissue types after immediate and delayed repair. Gene expression analysis revealed that 962, 588, and 3530 genes (median fragments per kilobase of transcript per million mapped reads (FPKM) = 25) were differentially expressed in macrophages, SC and distal stump, respectively, at day 5, and 273 and 462 genes (median FPKM = 22) were differentially expressed in macrophages and SC, respectively, at day 14 after repair (Supplemental Fig. 1a, b). Gene expression analysis also revealed downregulation (red) of immune response and angiogenesis in the distal stump 5 days after repair with concurrent upregulation (green) of neuron development and axon guidance (Supplemental Figs. 1c and 2).

**Fig. 2:**
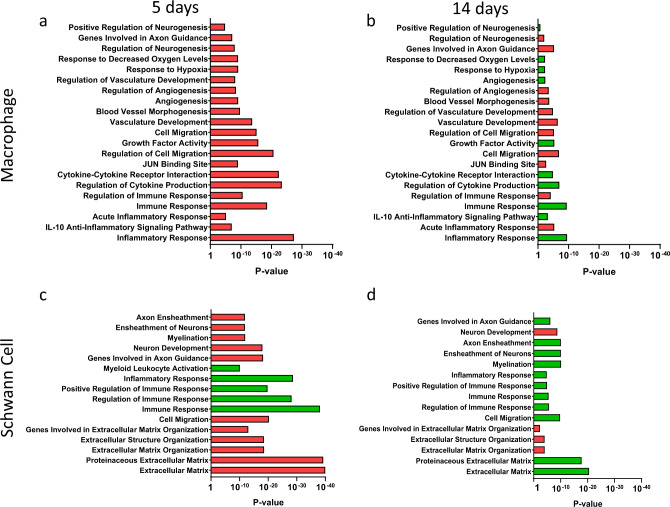
Delayed repair further delays regeneration. Downregulation (red) of immune response, angiogenesis, and IL10 signaling pathways identified in macrophages at day 5 was identified, followed by upregulation (green) of these biogroups at day 14. In SC, extracellular matrix formation, cell migration, and myelination were downregulated at day 5 compared to day 14 demonstrating the extension and progressive worsening of the repair process when repair is delayed. RNA sequencing data from delayed CP-TIB graft vs immediate CP-TIB graft for macrophages (**a**, **b**) and Schwann cells (**c**, **d**) after repair shows downregulation (red) or upregulation (green) of genes in biogroups at (**a**, **c**) day 5 or (**b**, **d**) day 14 after delayed repair. Cells isolated using FACS. *n* = 8 animals (4 male, 4 female) per group per time point.

Macrophages detect hypoxia within the regenerative bridge and are responsible for the formation of a polarized microvasculature which guides the migration of SC^19^. Analysis of differentially expressed (DE) genes into biological clusters (biogroups) identified downregulation of the immune response, the response to hypoxia and angiogenesis in macrophages at day 5 followed by upregulation of these biogroups at day 14 (Fig. 2a, b). These data indicate a lag in the macrophage response when repair is delayed. SCs, on the other hand, modulate the local immune response after injury^43,44^ and play an essential role in axon guidance, myelination and formation of an extracellular matrix. We identified the downregulation of extracellular matrix formation, cell migration and myelination in SC at day 5. The same biogroups were upregulated at day 14 (Fig. 2c, d) confirming the extension of the repair process in another key cell type the case of delayed repair.

**Fig. 3:**
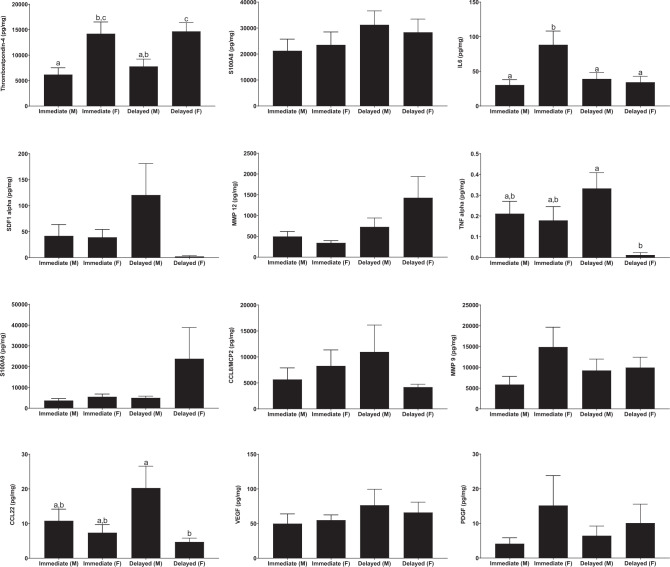
Delay in the repair of the nerve after peripheral nerve injury significantly alters the expression of inflammatory and ECM remodeling proteins. IL6, SDF1-alpha, and TNF-alpha were significantly reduced in female animals; whereas MMP-12 was significantly elevated in both sexes. Protein expression determined by Luminex assay and standardized to total protein level with a 5-mm inert silicone conduit inserted at the sciatic repair site. Significance was determined by ANOVA and. Groups not connected by the same letter are significantly different (Tukey post hoc test, *P* < 0.05). Letters not shown if ANOVA *P* > 0.05. *n* = 12 per group per sex.

We next determined the effect of delayed repair on protein expression. We identified significant alterations in inflammatory proteins known to be important in peripheral nerve injury (Fig. 3). IL6 collected from a short inert silicone conduit implanted at the graft site^35^ were significantly higher in female animals after acute repair than in other (*P* < 0.05, ANOVA with Tukey, *n* = 8–13 per group); and TS4 demonstrated significant differences in expression between sexes. In contrast, MMP-12, which is involved in the breakdown of extracellular matrix and tissue remodeling^45^ was significantly elevated after delayed repair in both male and female animals (*P* = 0.03, pooled sexes). These protein expression data support the altered inflammatory and extracellular matrix remodeling responses identified in our RNA-seq/FACS data.

### Delayed repair does not affect macrophage phagocytic function

**Fig. 4:**
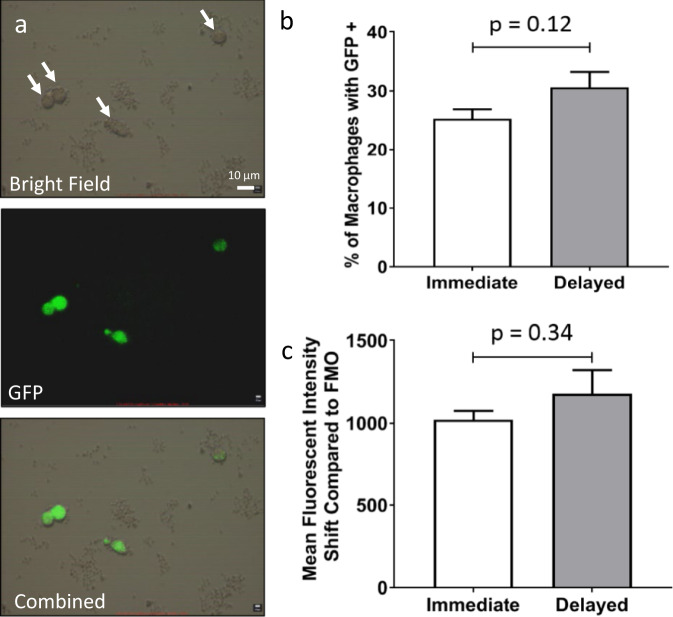
Delayed repair does not affect macrophage phagocytosis. Fluorescent microscopy indicates the presence of GFP signal after phagocytosis of bioparticles by macrophages (**a**) (white arrows). Macrophage phagocytic function was analyzed by GFP signal generated from phagocytosed bioparticles using FACS. No significant differences were observed in the percentage of macrophages with positive GFP signal (**b**) or the mean fluorescent intensity shift (**c**) between macrophages harvested from immediate and delay groups. No significant difference was observed between male and female animals (*t* test at *P* < 0.05, *n* = 12/group, 6 males, 6 females).

Macrophages are a major cell type in the first few days after injury, following neutrophil accumulation^19,46,47^, and phagocytose myelin debris^14,21,48^. To evaluate the phagocytic function of macrophages, the macrophages were isolated from the regenerative bridge using FACS. Macrophages were exposed to bioparticles which fluoresce when in contact with the phagocytosome (Fig. 4). No differences in phagocytosis by macrophages were detected between immediate and delayed repair groups (Fig. 4b, c).

### IL10 as a potential target for modulation of the macrophage response to delayed repair

Further examination of macrophage gene expression data at the peak of macrophage infiltration (5 days after repair) identified a mix of pro-inflammatory and anti-inflammatory genes known to be expressed by macrophages at the site of nerve repair (Supplemental Fig. 3)^48–52^. In prior work, we had identified IFNɣ as a key upstream regulator of temporal changes in macrophage gene expression^50^. As IFNɣ is downregulated by IL10, and IL10 was the anti-inflammatory gene with the highest differential expression (Supplemental Fig. 3a), we next examined the effect of IL10R deletion on macrophage gene expression in mice with a myeloid-specific deletion for IL10ra and demonstrated a significant increase in pro-inflammatory and angiogenic genes such as IL6, Saa3, and Cd31 when IL10ra was absent (Supplemental Fig. 3b). These data suggest that downregulation of IL10 in delayed repair may have a marked effect on macrophage expression of other inflammatory cytokines at the repair site.

### Immunomodulation with exogenous IL10 promotes macrophage recruitment and migration, promotes motor unit regeneration and axonal extension; polarizes macrophages toward an anti-inflammatory phenotype and promotes recovery when repair is delayed

**Fig. 5:**
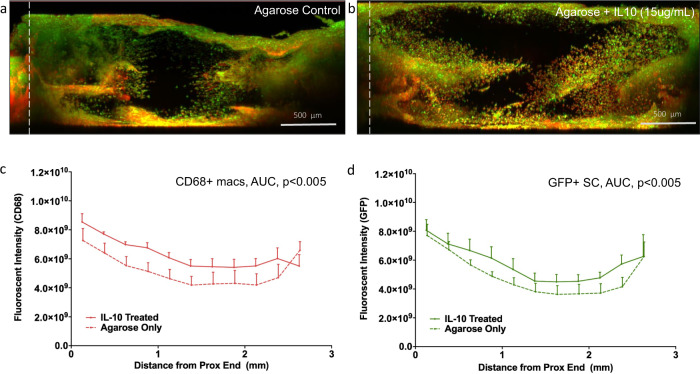
Transient immunomodulation of macrophage phenotype with exogenous IL10 promotes macrophage recruitment. Representative regenerative bridges from S100b GFP mice after immunohistochemistry staining with CD68 + macrophages (red) GFP + Schwann cells 10 days after 5-mm conduit repair augmented using agarose without (**a**) and with (**b**) IL10 (15 µg/mL) (white line indicates the location of the proximal stump). **c** Total number of recruited CD68 + macrophages (area under the curve, AUC) and SC (**d**) is increased in the presence of exogenous IL10 (*P* < 0.005). Sciatic nerve was transected in WT mice and repaired immediately with a 5 mm conduit filled with 0.7% agarose loaded with IL10 at 15 µg/mL or unloaded (negative control). Fluorescent intensity determined for contiguous 250 µm ROIs.

As IL10 was one of the most significantly downregulated genes in the macrophage population when repair is delayed, we next explored the potential of exogenous IL10 to modulate the early immune response at the site of nerve repair. S100 mice were also used to determine macrophage recruitment and SC migration within the regenerated nerve bridge 10 days (mid-stage) after repair a short 5-mm conduit^35^. Analysis of confocal images of the whole regenerative bridge indicated significantly higher recruitment of macrophages and their migration along the nerve bridge (CD68+, red) in the presence of exogenous IL10 (*P* < 0.005, Fig. 5). The Schwann cell migration along the regenerative bridge distal to the proximal stump (GFP+) was also increased especially at the center of the regenerative bridge (Fig. 5d).

**Fig. 6:**
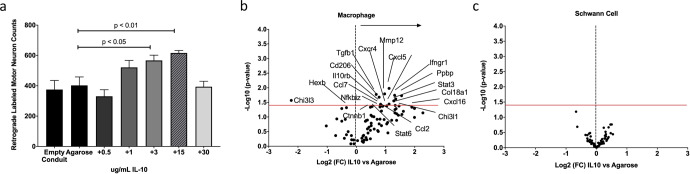
Transient immunomodulation produces a dose-dependent improvement in motor neuron regeneration. **a** Dose-dependent effect of exogenous IL10 in mice 8 weeks after repair. Number of retrograde-labeled regenerating MN were significantly elevated in the 3 µg/mL and 15 µg/mL IL10 groups (*P* < 0.05, *P* < 0.01, Dunnets vs agarose only control). **b** IL10 modulates local macrophage (**b**) but not Schwann cell (**c**) response. Gene expression (nanoString) of macrophages and Schwann cells isolated from the repair site by FACS 3 days after sciatic nerve repair in the presence (15 µg/mL) and absence (agarose only) of IL10. Significantly different gene expression above the horizontal red line representing false discovery rate (*P* <0.05, nSolver, Naonstring). **c** No differences were observed in SC gene expression at the same time point.

We next determined the does-response curve for IL10 at the site of peripheral nerve repair in mice. Exogenous IL10 was added to an agarose hydrogel within a short 5-mm gap defect^35^ and demonstrated a markedly dose-dependent effect; reaching a peak response at 15 µg/mL (*P* = 0.01, Fig. 6a) and then declining. We selected 0.7% agarose as a hydrogel delivery vehicle for IL10 as it is biocompatible and has minimal effects on motor unit regeneration^35,53^. No differences were observed between empty and agarose filled conduits as also seen in previous work^35^. A similar effect was seen in cranial tibial muscle weights (L:R ratio) although no significant differences were observed (Supplemental Information and Fig. 4). Therefore, 15 µg/mL IL10 mixed in 0.7% agarose was used for all subsequent experiments in mice.

To evaluate the effects of immunomodulation with exogenous IL10 on macrophagfe and SC gene expression FACS was used to isolate macrophages and SCs from S100 mice using a previously validated approach^38^ and gene expression for cell type evaluated against a custom panel of digitally barcoded probes (Nanostring^TM^)^34^. Exogenous IL10 promoted the expression of genes associated with anti-inflammatory response in macrophages, including Stat3, Stat6, IL35, Tgfb1, Cxcl16, and Chi3l1 (Fig. 6b, *P* < 0.05). Genes associated with ECM breakdown (Ccl7, Mmp12) and angiogenesis (Cxcl5, Cxcr4) also demonstrated significantly higher expression in macrophages after exogenous IL10 delivery. No significant differences were identified in Schwann cells gene expression (Fig. 6c). No sex effects were identified in either population.

**Fig. 7:**
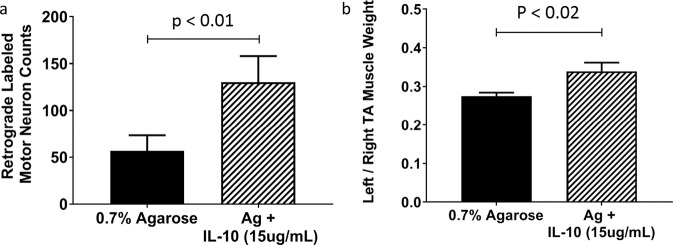
Exogenous IL10 significantly improves recovery of reinnervation after delayed repair. Number of regenerated motor neurons (**a**, *P* < 0.01) and left:right TA muscle mass (**b**, *P* < 0.02) significantly increased with transient immunomodulation of murine sciatic nerve repair with exogenous IL10 (15 µg/mL) vs agarose only control 8 weeks after repair. The tibial (TIB) nerve branch was transected in mice. After 8 weeks of delay, the proximal (common peroneal, CP) and distal (TIB) stumps were aligned and sutured 1 mm into a 5-mm silicone nerve conduit to create a noncritical defect (3 mm) in a CP-TIB graft. Retrograde labeling was conducted 8 weeks after repair. Significance set at *P* < 0.05 using *t* test.

Finally, to explore the therapeutic potential of exogenous IL10 after delayed repair, we used a CP-TIB repair paradigm. Exogenous IL10 improved recovery delayed repair demonstrated by the significant increase in the number of retrograde-labeled motor neurons (Fig. 7a, *P* < 0.01) and a significant increase in rennervated muscle weight (Fig. 7b, *P* < 0.02).

### Immunomodulation with exogenous IL10 promotes axonal growth and formation of neuromuscular junctions 8 weeks after transection and repair in rats

To validate these results in different species and begin to translate them, a subset of experiments were repeated in rats. For these experiments, transgenic rats expressing green fluorescent protein (GFP) under the Thy1 promotor (Thy1-GFP) in their peripheral axons were used^54,55^. These animals demonstrate a similar pattern of recovery after nerve injury to the WT background^56^.

**Fig. 8:**
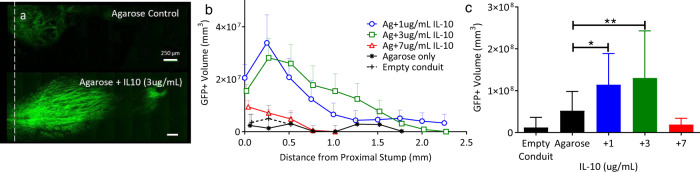
Transient immunomodulation in rats promotes dose-dependent axon extension. **a** Representative flattened z-stacks of regenerative bridges 21 days conduit repair. Bridges shown are agarose (ag) only or ag + IL10 (3 µg/mL). The White dashed line denotes the proximal stump. **b** GFP + axons per 250 μm contiguous ROI extending from the proximal stump. **c** Dose-dependent effect of exogenous IL10 with significantly increased axonal extension at 1 and 3 µg/mL (Dunnets to agarose only, *P* < 0.05) but not 7 µg/mL. GFP expression depicted as total GFP + axon volume. Data are mean and standard error (s.e.). *n* = 6–8 animals per group, male and female.

**Fig. 9:**
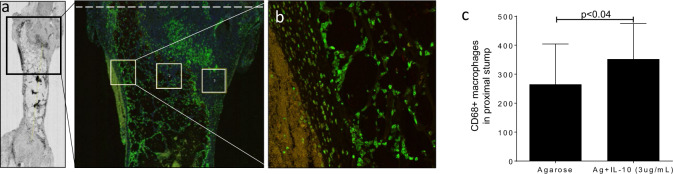
Transient immunomodulation in rats promotes macrophage recruitment. **a** Representative regenerative bridge after immunohistochemistry staining with CD68 + macrophages (green) 10 days after placement of a 10 mm conduit after sciatic transection. Counts obtained from three 500 µm × 500 µm regions of interest (ROI, white rectangles) per section (three sections/animal) per group. ROI placed on the periphery of the proximal stump of the regenerative bridge and centrally (white dashed line indicates the proximal stump). **b** Selected region of interest. Total number of CD68 + macrophages is increased (**c**) in the presence of exogenous IL10 (*P* < 0.04, *t* test).

**Fig. 10:**
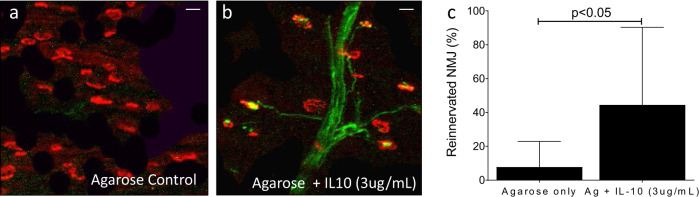
Immunomodulation promotes neuromuscular junction formation 8 weeks after injury. EDL muscle of GFP rats 8 weeks after sciatic conduit repair with exogenous IL10 (3 µg/mL) shows the increased formation of neuromuscular junctions (NMJ, *P* < 0.05). Representative images of terminal axons (green) innervating postsynaptic Acetylcholine receptors labeled with rhodamine-ɑ-bungarotoxin (red) conjugated to Alexa Fluor-594 in control (**a**) and treated groups (**b**). The proportion of innervated NMJ standardized to contralateral control indicates a significant increase in NMJ formation with exogenous IL10 (**c**, *P* = 0.049, two-tailed *t* test, *n* = 8 per group, 4 male, 4 female).

In these animals, as in mice, the response of axonal extension to exogenous recombinant rat IL10 demonstrated dose-dependent effects with a peak at 3 µg/mL for both GFP + axonal profiles per contiguous region of interest (ROI) and total GFP + volume within the 8 mm gap defect (Fig. 8a–c). We next evaluated macrophage recruitment (CD68+, green) to the regenerative bridge 21 days after injury, and we identified increase recruitment when exogenous IL10 was used at the time of repair (Fig. 9, *P* < 0.04). To determine recovery in a transection model, the most common type of PNI, rhodamine-ɑ-bungarotoxin conjugated to Alexa Fluor-594 was used to label postsynaptic terminals within EDL muscle^54^. Eight weeks after direct repair supported by subepineural injection with 50 µL of 0.7% agarose ±3 µg/mL IL10, the proportion of innervated neuromuscular junctions was significantly increased in the IL10 group (Fig. 10, *P* < 0.05).

## Discussion

We have found that delayed nerve repair produces a downregulated inflammatory response within macrophage and SC populations. This diminished response in these critical cell populations may contribute to poor functional outcomes when repair is delayed^3,5,6^ in addition to the loss of association between SC and their axons^2,6,7,10^ and alteration in extracellular matrix components in the distal degenerated nerve stump^2,7,9,10^. We demonstrated that pathways associated with angiogenesis, the immune response and axon guidance are downregulated early after delayed repair and that many of these pathways are upregulated later in the response.

Effective neuroregeneration after peripheral nerve injury requires a robust macrophage response; macrophages are the predominant cell type at the site of nerve repair in the first few days after injury and these cells are responsible for the formation of a polarized microvasculature which directs SC migration within the regenerative bridge^19–21,25,41^. Here, we demonstrated that immunomodulation of this macrophage response, occurring early in the repair process, can dramatically influence downstream regenerative events and lead to improved recovery. The selection of IL10 as a target for early immunomodulation was supported by both the downregulation of IL10 in macrophages following delayed repair in addition to casual pathway analysis of RNA sequencing data identifying IFNɣ, downregulated by IL10^49,52^, as a key upstream regulator of temporal changes in macrophage gene expression^34^.

Prior work has demonstrated that IL10 expression is upregulated in the distal stump following nerve crush injury reaching a peak at day 7 and returning to basal levels by day 14^51^ and that this is coincident with the migration of macrophages to the injury site. Although SC and fibroblasts also contribute to the IL10 expression^57^, the majority of expression is by macrophages^51,58,59^ peaking at day 7 after injury to reduce collagen level and scarring^60^ to promote myelination and neuroregeneration. However, macrophage-derived IL10 can have an inhibitory effect on the differentiation of neighboring cells into classically activated macrophages^61^ which may result in a self-regulation of macrophage populations at the site of injury and possibly by inhibiting further macrophage recruitment and debris removal.

In this study, we focused on nerve transection injury, as although less common than nerve crush, the recovery outcomes are significantly inferior to crush^62–64^. In the context of nerve crush, IL10 is upregulated in the distal segment reaching a peak of expression at day 7 and then returning to basal levels at day 14^51^.

We demonstrated that the addition of exogenous IL10 at the site of nerve injury polarizes the macrophages toward anti-inflammatory response and promotes macrophage recruitment, Moreover, we indicated a dose-dependent effect of exogenous IL10 in both mice and rats. Similar effects have been reported previously in which low dose of IL10 has resulted in better regeneration of damaged axons and greater myelination^58,60^ while the higher concentration had no beneficial effects on neural regeneration^58,65^. Immunomodulation with exogenous interleukin-4 (IL4) has also been shown to promote macrophage migration and axon extension at the site of nerve repair^66^.

Our data provide a clearer picture of such dose-dependency and the therapeutic potential of this approach in two different species. Previous studies have shown a direct correlation between monocyte chemoattractant protein (MCP1) and IL10^67^. MCP1 plays a critical role in innate immunity by directing the migration of monocytes into injury sites^67,68^. Despite the inhibitory effect of IL10 on INF and TNF, two key players in macrophage recruitment^51,65^, the upregulation of MCP1 production by IL10 can result in a higher level of macrophage recruitment into the injury site. In our study, immunofluorescence labeling indicates statistically significant higher macrophage recruitment to the site of nerve injury in the presence of exogenous IL10 in both mice and rats. The migration of macrophages and SCs from both proximal and distal stumps towards the middle of the nerve bridge in mice indicates the positive effect of exogenous IL10 in recruiting and migration of macrophages.

Although we show that SC gene expression is unaltered by the addition of exogenous IL10, we also demonstrated increased SC migration and axonal extension which suggests that the beneficial effects of exogenous IL10 may be mediated by the macrophage response to injury^19^. The role of macrophages after a delayed repair has not been previously evaluated. Most efforts to improve repair after nerve injury have focused on manipulating events occurring realtively late in the repair process, manipulating SC populations, and promoting axon extension^27–32^.

Delayed repair with a nerve graft is associated with poorer outcomes both experimentally and clinically. In a different experimental context, a prior crush injury or “conditioning lesion” 2 weeks prior to transection with immediate repair has been shown to increase the rate of axonal extension^69^.

This type of conditioning lesion produces a strong cell body response in axotomized remote dorsal root ganglion neurons^70^ including the release of inflammatory cytokines and activation of DRG resident macrophages^71,72^. More recent work has delineated differences in the inflammatory responses at the nerve injury site and the DRG in response to this type of conditioning lesion and this preactivated inflammatory state likely contributes to accelerated repair following a conditioning crush lesion. Analysis of the immune response to sciatic nerve injury identifies efferocytosis as a key mechanism of nerve debridement^47^.

Together our data demonstrate that macrophage gene expression, recruitment and migration at the site of nerve injury can be profoundly influenced by transiently altering the microenvironment at the site of repair with the addition of an exogenous cytokine; and that this produces significant downstream consequences for axonal extension and neuromuscular junction formation. We also showed that the effects of delayed repair on macrophage phenotype can be partially rescued through the addition of an exogenous ligand. These effects are induced by a cytokine with a very short duration of action which suggests that manipulation of the macrophage response early after injury or graft can lead to long-term improvements in outcome^73,74^.

## Methods

### Experimental model and subject details

Animal studies were performed in accordance with the PHS Policy on Humane Care and Use of Laboratory Animals, the NIH guide for Care and Use of Laboratory Animals, federal and state regulations, and was approved by the Cornell University Institutional Animal Care and Use Committee. Animals were brought into the research unit and given at least 5 days acclimatization period prior to any procedure. Daily record logs of medical procedures were maintained. Rodent cages were replaced weekly. Animals were on a 12/12 h light–dark cycle and allowed food and water ad libitum. Group housing prior to medical procedures provided socialization. ARRIVE guidelines for reporting in vivo experiments were used throughout^75^.

Experiments were conducted in mice and rats. *Mice*: The C57BL/6J (JAX # 0664) strain and mice expressing Green Fluorescent Protein (GFP) under the human S100β promoter (JAX #05621) were obtained from Jackson Laboratories. Mice were bred in-house in stable colonies, and genotypes were confirmed by PCR (Transnetyx, Cordova, TN). To generate IL10R^F/F cre+^ (experimental) and IL10R^F/F cre−^ (control) mice, Jax stock J028146; B6(SJL)-Il10ra^tm1.1Tlg^/J—also known as IL10Rα^flox^, was crossed with Jax stock J004781; B6.129P2-Lyz2^tm1(cre)Ifo^/J, then backcrossed to create litters with animals homozygous for the flox gene, and with (experimental) and without (control) the cre gene. Mouse genotypes from tissue biopsies were determined with specific probes designed for each gene by a commercial genotyping service (Transnetyx, Cordova, TN).

*Rats*. We assessed the effects of immunomodulation on axon extension and neuromuscular junction formation in rats expressing GFP under the Thy1 promoter in their axons. These rats were generated by pronuclear injection of the Thy1-GFP plasmid of the mouse line (BOR1-tg plasmid)^54^ and have similar motor and sensory neuronal architecture, over ground and skilled locomotion, muscle force, muscle weights and histomorphometric assessments to wild-type rats^56^.

#### Animal injury model

Animals were anesthetized with 3% isoflurane and maintained under anesthesia with 2% isoflurane and oxygen. Analgesia was provided by subcutaneous buprenorphine simbadol (0.1 mg/kg) injection pre-operatively and 24 h after surgery. All surgical procedures were performed using sterile microsurgical technique with an operating microscope (Superlux 40, Carl Zeiss).

To create the CP-TIB nerve graft, a freshly axotomized common peroneal (CP) nerve branch was grafted to the acutely or chronically denervated tibial (TIB). To mimic a delayed graft, the TIB nerve branch was transected 2–5 mm distal to the sciatic trifurcation and sutured to the quadriceps muscle. After 8 weeks of delay^7^, the proximal (CP) and distal (TIB) stumps were aligned and sutured into either end of a 5 mm silicone conduit (Tuzic, Siliclear tubing, 4.6 mm OD, 1.98 mm ID) with 10/0 ethilon sutures to produce the CP-TIB graft with an interposed silicone conduit for cell and protein isolation. Immediate repair was achieved similarly but with immediate CP-TIB cross-suture (graft).

### FACS for cell isolation

Mice were euthanized at 5 or 14 days (*n* = 8 per time point, 4 males and 4 females) after repair to describe the early and mid-immune responses to nerve injury. Macrophages and Schwann cells were collected for RNA sequencing using FACS as we have previously validated^38^ and described^34^.

In brief, the regenerative bridge was harvested within the conduit by transecting the proximal and distal nerve stumps 1 mm from the end of the conduit. The epineurial sutures were cut and the regenerative bridge was removed from the conduit and placed in a Petri dish with 1 mL RPMI-1640 (Corning) and was cut into 0.5 mm pieces. The tissues were then transferred to a 50 mL conical with 10 mL of digestion buffer. Single bridges from each animal were used and bridges were not pooled. The digestion buffer comprised of 3 mg/mL collagenase type I (Sigma), 1 mg/mL hyaluronidase (Sigma), and 0.5 mL of 1 mM HEPES in RPMI-1640. After 1 h digestion in a 37 °C water bath, tissues were strained through a 70-μm mesh strainer (BD Biosciences) to obtain a single-cell suspension. The cells were centrifuged at 300 × *g* for 10 min. The cell pellet was resuspended in 0.5% BSA (Sigma) in DPBS and cells were plated on a v-bottom 96-well plate (Nunc, Thermo Scientific) for antibody staining and FACS.

Cells were labeled for FACS for 45 min at 4 °C using species-specific antibodies to label macrophages and other immune cells^34,38^. Cells were washed two times after labeling. All wash steps were performed with 0.5% BSA in DPBS and propidium iodide (PI) was added after labeling as a viability marker. Prior to sorting, the nozzle, sheath, and sample lines were washed with RNAse Away (Ambion) for 15 min then flushed with preservative-free sheath solution (Biosure) for 2–3 min to remove RNases. A 100-μm ceramic nozzle (BD Biosciences), sheath at the pressure of 20 psi, flow rate <3 μL per second and acquisition rate of <3000 events per second were used as conditions optimized for neuronal cell sorting as previously described^76^. Cells were analyzed and sorted using FACS (BD Biosciences, AriaIII) and FACSDiva software (BD Biosciences version 6.1.3). The fluorochromes were excited with the instrument’s 405-, 488-, 532-, and 633-nm lasers. The appropriate detection filters were used. Compensation beads (OneComp, eBioscience) were used to set the compensation matrix. Fluorescence was determined by gating against appropriate controls (unstained, fluorescence minus one) on samples prepared in parallel. Gates were set such that less than 1% of positive events were recorded when acquiring the corresponding negative control.

Cells were gated on forward and side scatter areas for general cell size, forward scatter height and width to exclude doublets, forward scatter and PI to exclude dead cells, and F4/80 versus APC to remove cells in the dump channel (APC). Macrophages were defined as all viable single cells that were PI^−^ APC^−^ F4/80^+^ CD14^+^ CD16/32^+^ CD11b^+^. Schwann cells were defined as all viable single cells negative for all markers and showing GFP^+^ (PI^−^ APC^−^ F4/80^−^ CD14^−^ CD16/32^−^ CD11b^−^ GFP^+^). Antibodies used were as follows (antibody/probe, clone, fluorophore, distributor/cat no., specificity): CD16/32, 1:50, BV-605, BD Biosciences/563006, Pan macrophage; F4/80, 1:400, PE-Cy7, eBioscience/25-4801, Pan macrophage; CD11b, 1:200, Pacific Blue, BioLegend/101224, Pan macrophage; CD14, 1:100, PE, eBioscience/12-0141, Pan macrophage; Ly6G, 1:100, APC, eBioscience/17-9668, Neutrophils; Siglec F 1:64, APC, Miltenyi Biotec/130-102-241, eosinophils; CD19, 1:400, APC, eBioscience/17-0193, B lymphocytes; CD3e, 1:800, APC, eBioscience/17-0032, T lymphocytes; Thy1.2 1:800, APC, eBioscience/17/0902, fibroblasts; CD31, 1:200, APC, BD Biosciences/551262, endothelial cells; Ter119 1:200, APC, eBioscience/17-5921, Red blood cells; PI, 1:200, eBioscience, 00-6990, Viability. Dilutions were determined by titration. To assure specificity, purity checks were performed by re-analyzing a subset of sorted cells and only sorts with >80% enrichment were accepted. For each cell type, 1000 cells were sorted into 0.5% BSA in DPBS in RNase-free, lo-bind Eppendorf tubes (Zymogen). Sorted cells were centrifuged at 350 × *g* for 6 min. The supernatant was carefully removed, and the cells were sorted in 10 μL of RNase-free water at −80 °C until processed for RNA sequencing.

### RNA sequencing

#### RNA isolation

Total RNA from isolated macrophages and SC obtained from FACS was purified using Trizol (Thermo Fisher) according to the commercial protocol with the following additions: after the first phase separation, an additional chloroform extraction step of the aqueous layer in Phase-lock Gel heavy tubes (Quanta Biosciences); addition of 1–2 μL Glyco-blue (Thermo Fisher) immediately prior to isopropanol precipitation; two washes of the RNA pellet with 75% ethanol. RNA sample quality was confirmed using a Qubit3 (RNA HS kit; Thermo Fisher) to determine the concentration and with a Fragment Analyzer (Advanced Analytical) to determine RNA integrity.

#### RNA-seq library preparation for FACS-isolated cells

For macrophage and Schwann cells isolated by FACS, RNA from 1000 cells was amplified with the SMART-Seq v4 Ultra Low Input RNA Kit (Takara). Following Trizol purification, the RNA pellet was resuspended in 10.5 μL 1× reaction buffer. Half of the cDNA was used for PCR with 13 cycles of amplification yielding an average of 13 ng per sample for macrophages and 6 ng per sample for Schwann cells. TruSeq-barcoded libraries were prepared with the NEBNext Ultra II FS DNA Library Prep Kit (New England Biolabs) using 4 ng of amplified cDNA from macrophage samples and 1.5 ng from Schwann cell samples.

#### RNA-seq library preparation for tissue samples

Ribosomal RNA was subtracted by hybridization from 100 ng distal stump RNA samples using the RiboZero Magnetic Gold H/M/R Kit (Illumina) using a low-input protocol (Clontech). Following cleanup by precipitation, half of the rRNA-subtracted RNA was used to prepare TruSeq-barcoded libraries with the NEBNext Ultra II Directional RNA Library Prep Kit (New England Biolabs).

#### Illumina sequencing

Each RNA-seq library was quantified with a Qubit 2.0 (dsDNA HS kit; Thermo Fisher) and the size distribution was determined with a Fragment Analyzer (Advanced Analytical) prior to pooling equimolar amounts of each library for sequencing. Libraries were sequenced on a NextSeq500 instrument (Illumina) generating at least 20 M single-end 85 bp reads per library.

#### RNA-seq data analysis

Reads were trimmed for low quality and adapter sequences with cutadapt v1. (parameters: -m 50 -q 20 –a AGATCGGAAGAGCACACGTCTGAACTCCAG --match-read-wildcards). For the rRNA-subtracted distal stump libraries, residual rRNA-matching reads were removed with bowtie2 v2.2 (default mapping parameters, --un-gz <non-rRNA-reads.fastq.gz >). For gene expression analysis, reads were first mapped to the reference genome/transcriptome (mm10) using tophat v2.1 (parameters: --library-type=fr-firststrand --no-novel-juncs -G < UCSC_mm10_genes.gtf >). FPKM values and statistical analysis of differential gene expression were output from cufflinks v2.2 (cuffnorm/cuffdiff) (parameters: -C contrasts.txt --library-type=fr-firststrand as appropriate). Principal components analysis and heatmaps were generated with JMP v11 (SAS). Differentially expressed genes of each sample type (macrophages and SC) were further filtered to only include genes that has minimum CPM of 5 in more than five animals. The data was then analyzed in Correlation Engine (Illumina) to generate biogroups.

### Protein expression

Mice were euthanized 5 days after immediate and delayed repair. To generate an adequate amount of material for analysis, 8-mm conduits were used for this experiment. The biofluid inside the conduits was drawn using a 1-mL syringe and transferred into a Millepore tube with 0.22-µm filter (GV Durapore tube, Part# UFC30GV0S). The sample was washed with 100 µL PBS and then centrifuged at 10,000 × *g* for 2 min. The filters were removed, and the samples were transferred over ice and prepared for protein expression analysis using Luminex screening assay in a 12-plex panel based on the manufacturer’s protocol. The nonparametric Wilcoxon test was performed using JMP Pro 12 software (SAS Institute Inc., Cary, NC). Significance was set as *P* < 0.05 throughout.

### Macrophage phagocytosis assay

C57/BL6J were euthanized 5 days after immediate or delayed CP-TIB repair with a 5 mm conduit sciatic repair. The regenerative bridge was harvested within the conduit and digested for 1 h in 37 °C water bath in digestion buffer. The single-cell suspension was obtained by straining through a 70-μm mesh strainer (BD Biosciences). The cells were centrifuged at 300 × *g* for 10 min and then resuspended in 0.5% BSA (Sigma) in DPBS. The number of cells in each sample was counted using hemocytometer. The cells were then incubated for 1 h in 37 °C water bath with bioparticles (pHrodo™ Green Zymosan bioparticles, 75 particles per cell). The number of particles per cell was selected based on pilot titration study. After the incubation, the cells were washed two times with DPBS and were plated on a v-bottom 96-well plate (Nunc, Thermo Scientific) for antibody staining and FACS, as described^34,38^. Briefly, macrophages were identified using the four antibodies (F4/80, CD14, CD16/32, CD11b), and the phagocytosis performance of the macrophages was evaluated using the GFP signal related to phagocytosed bioparticles. The outcome measures were assessed using *t* test. Significance was set as *P* < 0.05 throughout.

### Promotion of recovery after delayed repair by exogenous IL10

Injury and delayed graft at 8 weeks was performed on C57/BL6J mice with an interposed 5-mm silicone conduit model. Conduits were filled with either 0.7% agarose loaded with 15 µg/mL of IL10, or 0.7% agarose only (control). An agarose concentration of 0.7% was chosen, as increasing agarose concentration has been previously found to be inversely related to extent and rate of neurite extension^53^.

Retrograde labeling was performed at 8 weeks after repair^35^.

Briefly, mice were anesthetized and the repaired nerve was transected 5 mm distal to the repair site. The nerve stump was soaked in 10% FluoroRuby in 2% DMSO for 1 h, rinsed, and replaced under the biceps femoris muscle. Mice were sacrificed 5 days later, perfused with chilled saline followed by 4% paraformaldehyde, and the spinal cord was harvested 5 mm cranial and 5 mm caudal to the L4-S3 region, which encompassed all retrograde-labeled motor neurons. Explanted cords were fixed in 4% paraformaldehyde overnight and optically cleared with increasing concentrations of tetrahydrofuran followed by dichloromethane, and finally dibenzyl ether^30^. Spinal cords were imaged at ×10 magnification with 6.4 µm z-stacks using a confocal microscope (Zeiss 510, Thornwood, NY; 561 nm). The entire motor neuron pool of all labeled cells was counted by a single blinded observer using a customized automated cell counting program in MATLAB (Release 2015a, The MathWorks, Inc., Natick, MA) based on 3D Laplacian Gaussian object detection.

To determine the effect of exogenous IL10 to rescue the deleterious effects of delayed nerve graft on regeneration of motor units. The number of regenerated motor neurons in the spinal cord was compared between agarose only (control) and agarose with IL10 (treatment) groups using two-sided *t* test with significance set as *P* < 0.05.

### Macrophage recruitment and schwann cell migration in murine regenerative bridge

S100β eGFP mice were euthanized at 10 days after repair (for both treatment groups) to determine macrophage recruitment and SC migration. The regenerative bridge was harvested within the conduit by transecting the proximal and distal sciatic nerve stumps 1 mm from the end of the 5-mm conduit. The epineurial sutures were cut and the regenerative bridge was removed from the conduit and fixed overnight in 4% paraformaldehyde. To stain macrophages, the bridge was then incubated in goat-blocking serum and casein mixture for 5 h. The sample was washed in PBS and then incubated in macrophage-specific primary antibody (1:50 CD68, Abcam) overnight. After the wash step, the regenerative bridge was transferred into secondary antibody solution (1:200 biotinylated goat anti-rabbit IgG, Vector Laboratories) and incubated overnight. Sample was washed again in PBS and transferred into Texas-Red conjugate solution (1:200, Life Technologies) and incubated overnight. The sample was then washed and incubated in DAPI solution (1:1000) overnight. After the final wash in PBS, the sample was immersed into DIX solution (a mixture of n-methyl-d-glucamine, diatrizoic acid, 60% iodixanol and 2% sodium azide) to clear the tissue. All the wash steps were done twice in PBS over a rocking table for 30 min each. All the incubation steps were done at room temperature on a turning wheel at 20–40 rpm.

Samples were then mounted on slides while immersed in DIX solution and the entire regenerative bridge was imaged using tiled, Z stack imaging (Zeiss 710 confocal). The macrophage and SC presence and distribution within the regenerative bridge was quantified by the summation of the fluorescent intensity of each channel (GFP green: SC, Texas red: macrophage, DAPI blue: all cells) at 250 µm intervals from the distal end of the proximal stump using ImageJ. Results were analyzed with group—agarose only (control) or agarose with IL10 (15 µg/mL) concealed to the observer. Differences in total numbers of macrophages were determined using two-tailed *t* test with *P* < 0.05.

### Axon extension and formation of neuromuscular junctions in rats

We assessed axon extension after acute injury and the formation of neuromuscular junctions (NMJ) at 8 weeks in Thy1-GFP rats. Rats were anesthetized with 5% isoflurane, and maintained under anesthesia with 1–2% isoflurane. Analgesia was provided by Meloxicam (2 mg/kg) pre-operatively and 24 h post-operatively. The sciatic nerve was exposed and a 10-mm silicone conduit placed between the transected nerve ends and sutured with single 10/0 sutures to create an 8 mm defect. The conduits were either filled with 0.7% agarose hydrogel, or three different concentrations of rat-specific IL10 (1 µg/mL, 3 µg/mL, 7 µg/mL, recombinant rat IL10, Peprotech, part #400-19) with 0.7% agarose as a carrier. The muscle and skin were re-apposed using 6-0 Ethilon sutures. Body temperature was maintained by an electrical heating pad placed below rat within the surgical field; body temperature and heating pad temperature were monitored to maintain physiologic body temperature. All surgeons were blinded to treatment groups during surgical procedures and tissue harvests.

To determine axon extension after injury, five groups of animals (IL10 at 1, 3, or 7 µg/mL in 0.7% agarose, unloaded agarose or empty conduit, *n* = 6–8 animals/group, male and female) were allowed to recover for 21 days. Animals were then anesthetized and euthanized (pentobarbital (250 mg/mL, 4 mL/kg), and the regenerative nerve bridge was harvested. Bridges were placed in a well with PBS and imaged using confocal microscope (Zeiss LSM510 confocal). One spectral window for the emission profile of GFP (488 nm) and the entire extension of the regenerative bridge was imaged using tiled, Z stack images. Axonal outgrowth from the distal stump was quantified by evaluating axonal regenerative profiles extending through contiguous regions of interest (ROI) at 250-µm intervals from the distal end of the proximal stump using BitPlane (Imaris) and calculating GFP + volumes for each ROI. Differences between groups were determined by evaluating GFP + volume and the interaction between dose and contiguous ROI in a mixed-effect model, in the same group of animals.

To determine the effect of exogenous IL10 on neuromuscular junction formation, two groups of animals (*n* = 8/group) underwent sciatic transection and a silicone conduit repair (10 mm) filled with 3 µg/mL IL10 in 0.7% agarose or agarose only (negative control). Animals were euthanized at 8 weeks NMJ formation in the extensor digitorum longus (EDL) muscle was assessed for terminal axons (green) innervating postsynaptic acetylcholine receptors (AChRs), labeled with rhodamine-ɑ-bungarotoxin (red) conjugated to Alexa Fluor-594^54^. Briefly, the EDL muscles were harvested bilaterally for whole-mount confocal microscopy. The muscles were dissected into four separate muscle bellies to create thinner samples. The muscles were placed on Pyrex resin-coated dishes and stained with rhodamine alpha-bungarotoxin conjugated to Alexa Fluor-594 (2.5 µg/mL; Thermo Fischer Scientific, CA) at room temperature for 30 min to label the acetylcholine receptors in the postsynaptic membrane. The muscles were rinsed with phosphate-buffered saline and mounted under a coverslip for confocal microscopy. The concentration of alpha-bungarotoxin conjugate was validated using a dose curve in untreated GFP rats. The muscles were imaged using a Zeiss LSM510 confocal microscope. Two spectral windows using the Zeiss Zen image software were used: one using the emission profile of Alexa Fluor-594 (590 nm) and the emission profile of GFP (488 nm). To evaluate the thicker muscle samples, four equidistant z-series (stack) were taken across the muscle sample to acquire images of a minimum of 100 motor end plates. The observer was blinded to the treatment group at the time of acquisition of imaging and during image analysis. The images were used to count the number of innervated and non-innervated motor end plates; innervation was determined by the extension of a GFP-expressing axon to a positively labeled motor end plate. The proportion of innervated NMJ was standardized to each contralateral control.

### Reporting summary

Further information on research design is available in the Nature Research Reporting Summary linked to this article.

## Supplementary information

Supplemental Figures

Reporting Summary

## Data Availability

RNA sequencing data are available at the Gene Expression Omnibus (GEO) hosted by the National Institutes of Health (https://www.ncbi.nlm.nih.gov/geo/info/submission.html): Accession number GSE217172. Additional datasets generated during and/or analyzed during this study are available from the corresponding author upon reasonable request.

## Code availability

All code used to process the RNA sequencing and other data is available on request from the corresponding author.
