# Supplementary material for: Delay modulates the immune response to nerve repair
Source: NPJ Regen Med. 2023 Feb 27;8:12. doi: 10.1038/s41536-023-00285-4 (PMC9970988; doi:10.1038/s41536-023-00285-4)
Supplement: Supplementary file 1 — Supplemental Figures [file 41536_2023_285_MOESM1_ESM.pdf]

Supplementary Figures

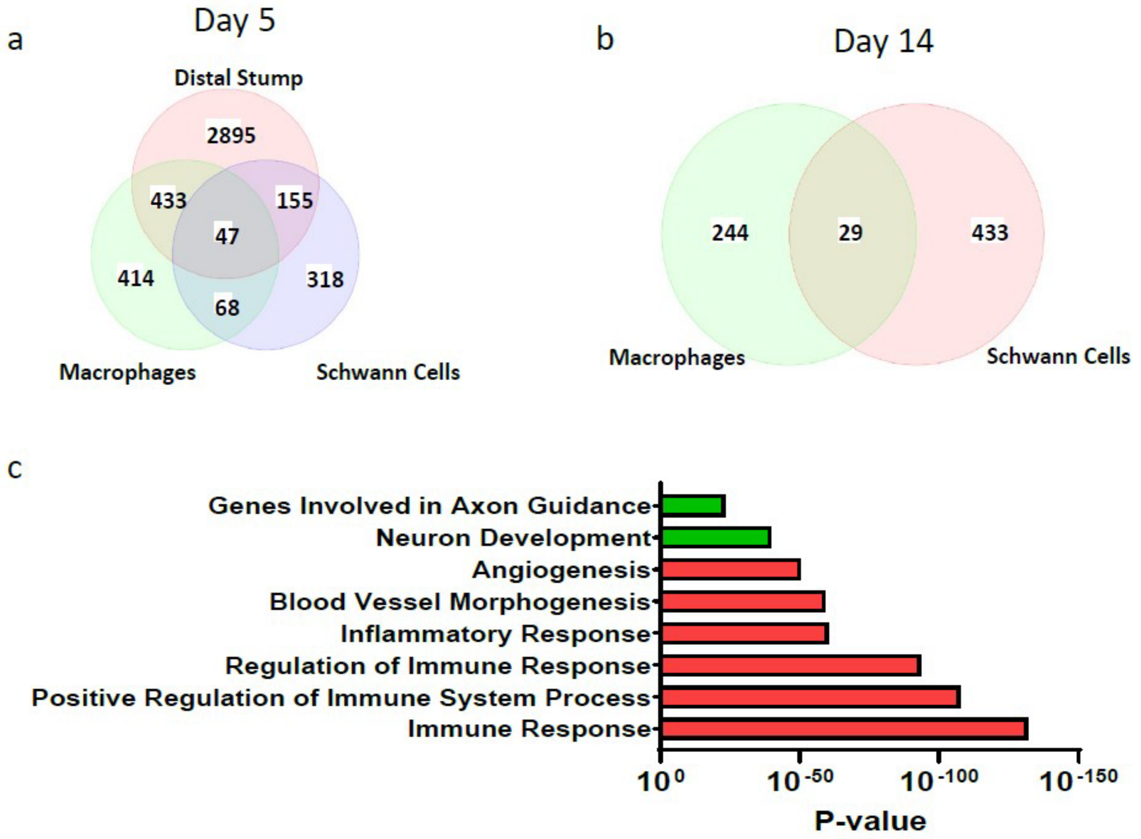

Supplemental Figure 1. Delay in the repair of the nerve after peripheral nerve injury results in impaired regeneration at distal stump. (a,b) Numbers of differentially expressed genes between immediate and delayed repair in the distal stump and in, macrophage and Schwann cell populations at day 5; and in mac and SC populations at day 14 after injury. (c) RNA sequencing results (delay repair vs immediate injury and repair) of the distal stump after repair shows downregulation (red) of immune response and angiogenesis in distal stump 5 days after repair (n=8 per group, 4 male and 4 female). Pathways associated with axon guidance and neuron development were upregulated in the distal nerve stump 5 days after repair. Distal nerve stumps were not evaluated at 14 days.

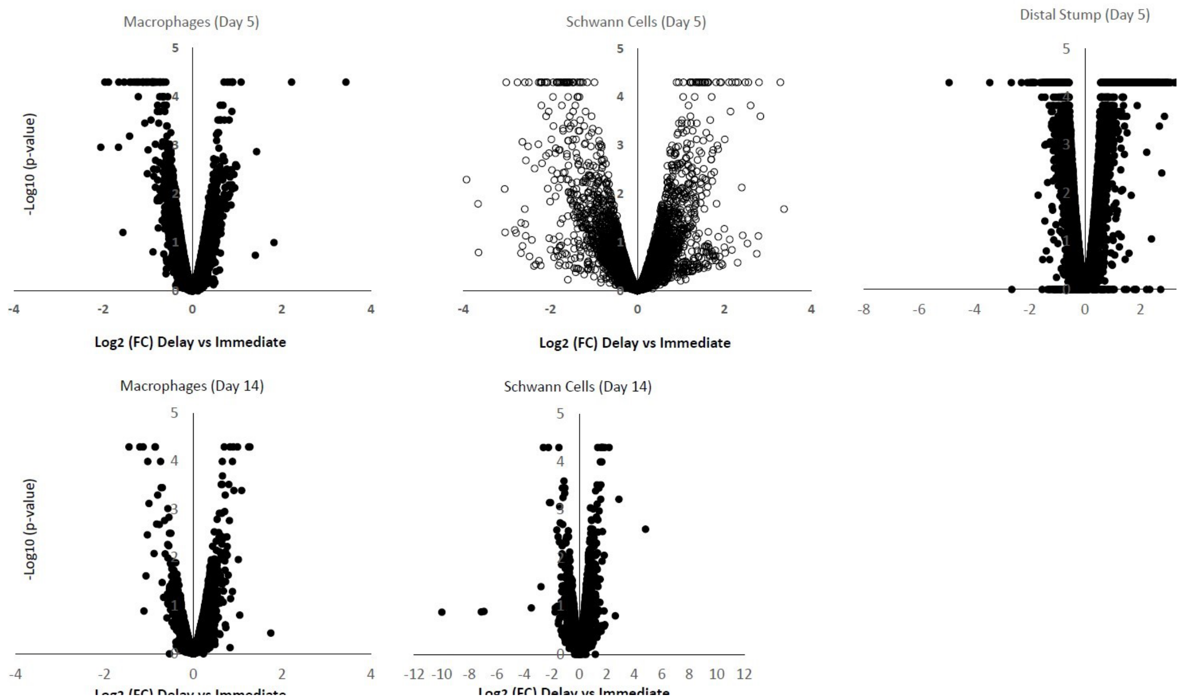

Supplemental Figure 2. Volcano plots of differentially expressed (DE) genes. Demonstrating log 2 foldchange (FC) for delayed and immediate repair groups. Data shown for mac, SC and distal stump 5 days after repair and mac and SC 14 days after repair. Animals as figure S1.

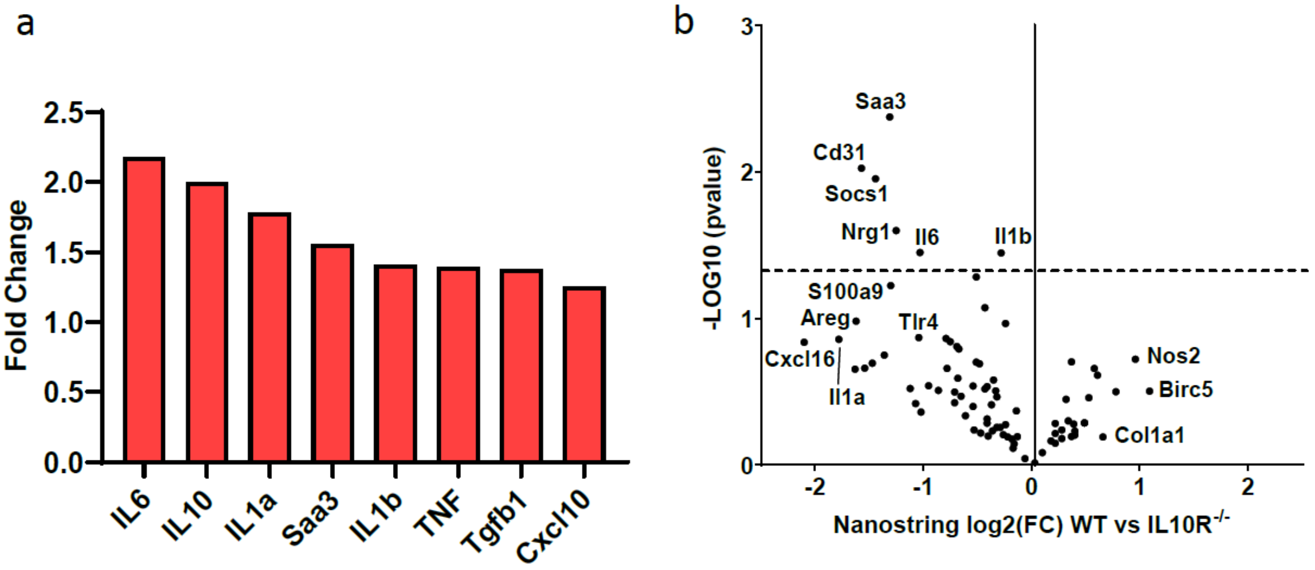

Supplemental Figure 3. IL10 is a key upstream regulator of immune response and alters expression of pro-inflammatory genes by macrophages after injury. Ranked fold change in immune response related genes obtained from RNA sequencing results in WT mice showing ranked relative downregulation of single genes following delayed repair (a, animals as figure S1.).(b) Effect of IL10ra deletion on macrophage gene expression 3 days after immediate repair demonstrating significant increases in expression of pro-inflammatory genes in macrophage population. P-values were obtained from nSolver software, with the false-discovery rate corrected level of significance indicated by the horizontal dotted line. Significance set at $p < 0.05$. Macrophages were sorted from sciatic nerves of IL10ra-/- and WT mice that were transected and repaired with an empty conduit 3 days before harvest n=6 per group, 3 males and 3 females).

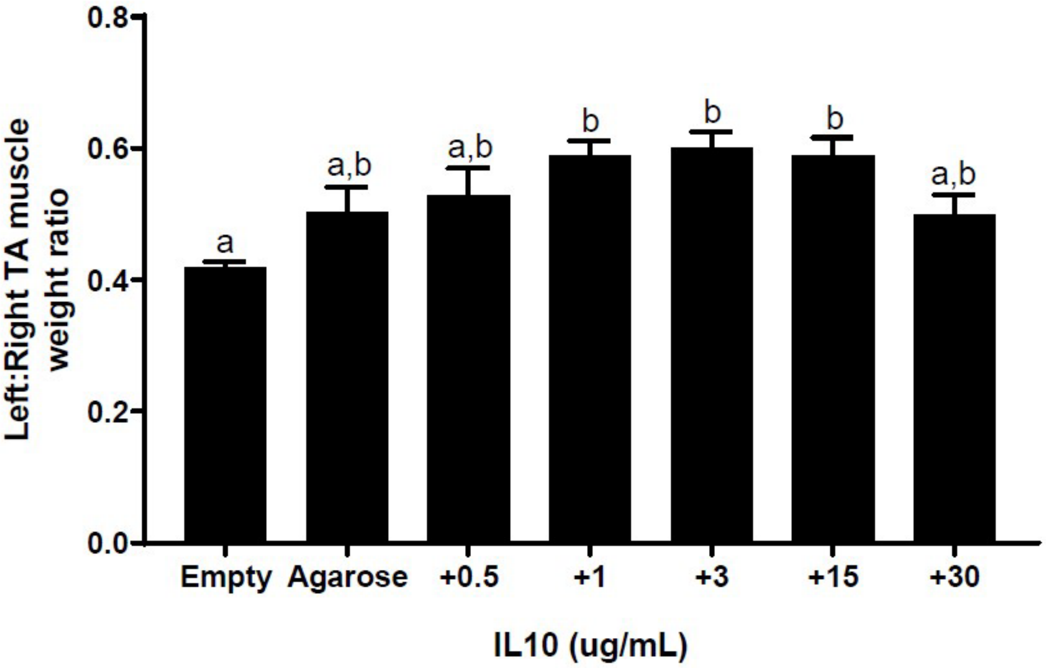

Supplemental Figure 4. Exogenous IL10 creates a dose-dependent change in Tibialis Anterior (TA) muscle weight ratio. Left and right muscle weight obtained from the animals described in figure 6a. Sciatic nerve was transected in WT mice and repaired immediately with a 5mm conduit filled with 0.7% agarose loaded with IL-10 at 0.5, 1,3,15 or 30μg/mL, unloaded (negative control), or empty conduit (additional negative control) (n=5-8 mice per group). The ration of left TA muscle weight (injured) over the right TA muscle weight (uninjured) at eight weeks demonstrates a similar improvement trend compared to motor neuron count by exogenous IL10 delivery (ANOVA, n=5-8 per group)
